# Effects of postmortem positional changes on conjunctival petechiae

**DOI:** 10.1007/s12024-018-0032-5

**Published:** 2018-11-03

**Authors:** Tomoya Ikeda, Naoto Tani, Yayoi Aoki, Alissa Shida, Fumiya Morioka, Shigeki Oritani, Takaki Ishikawa

**Affiliations:** 1 Department of Legal Medicine, Osaka City University Medical School, Asahi-machi 1-4-3, Abeno, Osaka, 545-8585 Japan; 2 Forensic Autopsy Section, Medico-legal Consultation and Postmortem Investigation Support Center, c/o Department of Legal Medicine, Osaka City University Medical School, Asahi-machi 1-4-3, Abeno, Osaka, 545-8585 Japan

**Keywords:** Petechiae, Palpebral conjunctivae, Body position, Body mass index, Cause of death, Forensic

## Abstract

The present study aimed to determine whether postmortem period, physical constitution, cause of death, and cardiopulmonary resuscitation are associated with positional changes in the postmortem appearance of conjunctival petechiae. We retrospectively investigated serial forensic autopsies from 6 h to 6 days postmortem (*n* = 442; male, 303; female, 139; median age, 62 years; range, 0–100 years). The causes of death were sharp instrument injury, blunt force trauma, fire, intoxication, asphyxia, drowning, hypothermia, hyperthermia, acute heart failure, and natural causes. Of these, 28 (male, *n* = 18; female, *n* = 10) were aged ≤5 years. Palpebral conjunctival petechiae were initially assessed at autopsy in supine bodies, then reassessed in prone bodies after 30 min. Among 414 bodies, 291 (70.2%) and 123 (29.7%) who were aged between 6 and 100 years, and 18 (64.2%) and 10 (35.7%) aged <5 years at the time of death, were discovered in the supine and prone positions, respectively. The amounts of petechiae increased within 1.5 days postmortem, but not in those discovered in the prone position. The rates at which petechiae increased were higher in supine overweight bodies (BMI ≥ 25.0) and in those who were discovered supine and had died of asphyxia or drowning (37.5%). Cardiopulmonary resuscitation for bodies discovered in the supine and prone positions did not statistically affect the occurrence of petechiae. Several postmortem factors can cause hypostatic blood redistribution that manifests as increased amounts of petechiae in the palpebral conjunctivae.

## Introduction

Conjunctival petechiae comprise an important forensic indicator of mechanical compression of the jugular veins caused by external force to the neck [1]. Consensus in the literature suggests that the pathogenesis of conjunctival petechiae is related to the combined effects of increased cephalic venous pressure and hypoxic damage to endothelial cells [2, 3]. However, the occurrence of petechiae does not only depend on external force to the neck, and it does change posthumously. Body position and other factors at death can influence the occurrence of conjunctival petechiae, which can also develop postmortem due to intravascular hypostatic pressure affecting the head and neck [1, 4–7]. To discriminate postmortem from antemortem petechiae is difficult. As a result, more than just dynamic force to the neck should be considered when discussing the occurrence of conjunctival petechiae [6].

The present study aimed to identify factors that were involved in the occurrence of conjunctival petechiae in 442 forensic autopsies. Factors included postmortem changes in body position, elapsed time after death, body mass index (BMI), cause of death and resuscitation.

## Materials and methods

Among 442 bodies found between 6 h to 6 days postmortem, 303 were male, 139 were female, and the median age was 62 (range, 0–100) years. Inclusion criteria were defined as the position of the body at the time of discovery (supine, *n* = 309; prone, *n* = 133). Tables 1 (age > 5 years) and 2 (< 5 years) show the causes of death that were determined based on the findings of complete autopsies, as well as the findings of macromorphological, micropathological, and toxicological examinations. The most prevalent cause of death among individuals aged >5 years who were discovered in the supine or prone position was blunt force trauma (*n* = 105 male and *n* = 36 female), followed by natural causes (*n* = 35 male and *n* = 15 female), fire (*n* = 30 male and *n* = 13 female) and intoxication (*n* = 28 male and *n* = 17 female). The remainder died of asphyxia (*n* = 19 male and *n* = 9 female), acute heart failure (*n* = 17 male and *n* = 7 female), sharp instrument injury 25 (*n* = 21male and *n* = 4 female), drowning (*n* = 8 male and *n* = 13 female), hyperthermia (*n* = 17 male and *n* = 3 female) and hypothermia (*n* = 11 male and *n* = 6 female).

**Table 1:** Case profiles of individuals aged >5 years at the time of death

Cause of death	Sharp instrument injury	Blunt force trauma	Fire	Intoxication	Asphyxia	Drowning	Hypothermia	Hyperthermia	Acute heart failure	Natural causes (others)
Supine position at time of discovery (*n* = 291)										
N	21	105	30	28	19	8	11	17	17	35
Age (y)	27–91 (60)	15–100 (62)	28–97 (69)	20–96 (47)	9–84 (54)	65–96 (78)	44–91 (61)	24–95 (67)	25–88 (65)	26–93 (67)
Sex (male/female)	12/9	79/26	17/13	19/9	13/6	6/2	9/2	11/6	13/4	22/13
Body mass index	17.2–28.9 (22.7)	12.0–49.4 (22.2)	14.3–40.7 (22.3)	14.8–35.4 (23.8)	14.0–30.8 (20.1)	16.1–23.5 (21.0)	12.0–21.8 (16.4)	13.0–25.3 (19.4)	14.2–29.7 (21.9)	11.6–37.4 (21.1)
Postmortem period (days)	0.5–3 (1.5)	0.5–14 (1.5)	0.5–2 (1)	0.5–10 (2)	1–4 (1.5)	0.5–2.5 (1.5)	1–30 (6)	1–10 (2.5)	0.5–20 (2.5)	0.5–7 (2)
Medical care	4	53	10	3	3	1	0	2	0	3
Prone position at time of discovery (*n* = 123)										
Number	4	36	13	17	9	13	6	3	7	15
Age (y)	44–70 (56)	18–79 (52)	47–73 (63)	20–60 (40)	23–68 (49)	19–80 (55)	47–84 (75)	63–71 (68)	16–67 (42)	11–80 (55)
Sex (male/female)	4/0	28/8	9/4	10/7	5/4	6/7	4/2	2/1	6/1	10/5
Body mass index	19.8–25.0 (22.5)	14.2–35.8 (21.9)	14.3–35.5 (20.7)	16.7–29.0 (22.4)	17.2–25.4 (21.1)	17.9–27.4 (21.7)	17.2–25.6 (19.3)	18.6–26.0 (21.1)	17.7–36.8 (24.4)	15.6–35.6 (22.9)
Postmortem period (days)	1–4 (3)	0.5–7 (1.5)	0.5–4 (1)	1–14 (3)	0.5–7 (2)	0.5–3 (2)	1–10 (4)	1–4 (2.5)	0.5–20 (3)	0.5–5 (1.5)
Medical care	1	11	4	0	2	0	0	0	1	0

Among 16 individuals aged <5 years who were discovered in the supine or prone position, natural causes other than acute heart failure comprised the most prevalent cause of death (*n* = 10 male and *n* = 6 female), followed by blunt force trauma (*n* = 5 male and *n* = 1 female), asphyxia (*n* = 2 male), and acute heart failure (*n* = 1 male and *n* = 1feamle). The remainder died of intoxication (*n* = 1 female) and hyperthermia (*n* = 1 female). No one aged <5 years died due to fire, drowning or hypothermia.

Exclusion criteria comprised finding different amounts of petechiae in the right and left conjunctivae, being discovered in the lateral decubitus position, and the loss of conjunctivae and eyelids due to environmental causes such as insects and putrefaction.

Palpebral conjunctivae were ectropionized from bodies at the start of autopsies, then congestion, and the presence and number of petechiae were documented. The amount of petechiae in the upper conjunctiva was classified as numerous (> 10), several (< 10), or absent. Bodies were then placed in the prone position in an autopsy room for 30 min at a temperature of 15 °C, and palpebral conjunctival petechiae were reassessed after returning the bodies to the supine position. Body mass index or the Kaup index, postmortem period, cause of death as reported on the death certificate, and preceding resuscitation efforts were documented as potential covariates.

**Table 2:** Case profiles of children aged <5 years at the time of death

Cause of death	Sharp instrument injury	Blunt force trauma	Fire	Intoxication	Asphyxia	Drowning	Hypothermia	Hyperthermia	Acute heart failure	Natural causes (others)
Supine position at time of discovery (*n* = 18)										
N	0	5	0	0	2	0	0	0	1	10
Age (y)	–	0–1 (0)	–	–	0, 3	–	–	–	0	0–1 (0)
Sex (male/female)	–	4/1	–	–	1/1	–	–	–	1/0	5/5
Kaup index	–	13.1–20.7 (17.6)	–	–	13.7, 15.5 (14.6)	–	–	–	17.8	11.4–17.4 (14.6)
Postmortem period (days)	–	1–2 (1.4)	–	–	1.5–2 (1.6)	–	–	–	1	1–4 (1.1)
Medical care	–	4	–	–	0	–	–	–	2	2
Prone position at time of discovery (*n* = 10)										
Number	0	1	0	1	0	0	0	1	1	6
Age (y)	–	4	–	0	–	–	–	0	0	0–4 (1)
Sex (male/female)	–	1/0	–	0/1	–	–	–	0/1	1/0	5/1
Kaup index	–	9.8	–	13.3	–	–	–	18.4	15.3	11.3–18.0 (14.6)
Postmortem period (days)	–	2	–	3	–	–	–	2	1.5	0.5–2 (1)
Medical care	–	0	–	0	–	–	–	0	0	0

### Statistical analysis

Variables were selected using backwards multivariate generalized estimating equations analyses with outcome variables distributed according to Poisson. Odds ratios (ORs) with 95% confidence intervals (CIs) were calculated for statistically significant risk factors. Nominal *p* values are reported without correction for multiplicity. Values with two-sided *p* < 0.05 were considered significant. Data were statistically analyzed using SPSS, Version 19.0.0 (IBM Corp., Armonk, NY, USA).

## Results

### Supine position

Among 309 (69.9%) of 442 bodies that were discovered in the supine position, 291 were aged >5 and 18 were aged <5 years.

### Appearance of petechiae based on postmortem period

Based on postmortem periods of about <12, 12–24, >24–36, >36–48, >48–60, >60–72, and > 72 h, petechiae appeared in approximately 100% (5/5), 22.8% (21/92), 17.2% (15/87), 9.8% (4/41), 11.1% (1/9), 0.0% (0/14), and 0.0% (0/43), respectively, of the bodies of individuals that were aged >5 years at the time of death. Petechiae were obvious within 1.5 days, compared with >1.5 days postmortem. Petechiae did not appear in the bodies of children aged <5 years at any time.

### Appearance ratios of petechiae based on BMI and Kaup index for individuals aged >5 and < 5 years, respectively

We classified BMI < 18.5 (*n* = 70), 18.5 to <25.0 (*n* = 159), 25.0–30.0 (*n* = 47) and BMI > 30.0 (*n* = 15) in those aged >5 years as malnutrition, normal weight, overweight and obesity according to World Health Organization standards.

Table 3 shows that petechiae increased in individuals that were classified as being overweight (29.8%) and having obesity (20.0%), compared with malnutrition (8.6%) and normal weight (14.5%) individuals.

**Table 3:** Changes in palpebral conjunctival petechiae according to body mass index in individuals aged >5 years and found in supine position

Ratios of petechial hemorrhage before changing position (*n* = 291)				
BMI	<18.5	18.5- < 25.0	25.0- < 30.0	30.0–34.99
N	70	159	47	15
Numerous (>10)	11.4% (8/70)	27.0% (43/159)	31.9% (15/47)	60.0% (9/15)
Several (<10)	20.0% (14/70)	31.4% (50/159)	29.8% (14/47)	13.3% (2/15)
None	68.6% (48/70)	41.5% (66/159)	38.3% (18/47)	26.7% (4/15)
Ratios of petechial hemorrhage after changing position				
Numerous (>10)	12.9% (9/70)	31.4% (50/159)	42.6% (20/47)	60.0% (9/15)
Several (<10)	22.9% (16/70)	31.4% (50/159)	26.5% (12/47)	20.0% (3/15)
None	64.3% (45/70)	37.1% (59/159)	31.9% (15/47)	20.0% (3/15)
Rates of increases in petechial hemorrhage				
	8.6% (6/70)	14.5% (23/159)	29.8% (14/47)	20.0% (3/15)

The prevalences of numerous and several petechiae before changing position based on BMI were 68.6% and 41.5% among bodies classified according to BMI as malnutrition and normal weight, respectively, and that of absent and numerous petechiae was 60.0% among those with obesity.

Prevalences after changing position according to body mass classification were as follows: malnutrition: no petechiae 64.3%; normal weight: numerous 31.4%; several 31.4% and none 37.1%; overweight: numerous 42.6%; obesity: numerous 60.0%.

We classified children aged <5 years with Kaup indexes of <13 as malnutrition (*n* = 2), 13 to <15 as thin (*n* = 6), 15 to <19 as normal weight (*n* = 9), and > 19 as overnutrition (*n* = 1). Several petechiae were found only in 33.3% of children with normal weight. However, petechiae were not affected after changing position (Table 4).

**Table 4:** Changes in palpebral conjunctival petechiae according to Kaup Index in children aged <5 years and found in supine position

Ratios of petechial hemorrhage before changing position (*n* = 18)				
Kaup Index	<13.0	13.0- < 15.0	15.0–19.0	>19.0
N	2	6	9	1
Numerous (>10)	0% (0/2)	0% (0/6)	0% (0/9)	0% (0/1)
Several (<10)	0% (0/2)	0% (0/6)	33.3% (3/9)	0% (0/1)
None	100% (2/2)	100% (6/6)	66.7% (6/9)	100% (1/1)
Ratios of petechial hemorrhage after changing position				
Numerous (>10)	0% (0/2)	0% (0/6)	0% (0/9)	0% (0/1)
Several (<10)	0% (0/2)	0% (0/6)	33.3% (3/9)	0% (0/1)
None	100% (2/2)	100% (6/6)	66.7% (6/9)	100% (1/1)
Rates of increases in petechial hemorrhage				
	0% (0/2)	0% (0/6)	0% (0/9)	0% (0/1)

### Appearance of petechiae according to cause of death

Table 5 indicates that the rates of numerous and several petechiae before changing the position of the body were high in individuals aged >5 years who died due to asphyxia (78.9% and 15.8%, respectively), fire (40.0% and 10.0%, respectively) and intoxication (32.1% and 21.4%, respectively). Rates of several petechiae were low for hypothermia (0.0% and 45.5%, respectively) and hyperthermia (5.9% and 23.5%, respectively). Other causes of death including sharp instrument injury, blunt force trauma, drowning, acute heart failure and other natural causes, were associated with moderate rates of petechiae.

**Table 5:** Changes in palpebral conjunctival petechiae according to cause of death in individuals aged >5 years and found in supine position

Cause of death	Sharp instrument injury	Blunt force trauma	Fire	Intoxication	Asphyxia	Drowning	Hypothermia	Hyperthermia	Acute heart failure	Natural causes (others)
Ratios of petechial hemorrhage before changing position (*n* = 291)										
N	21	105	30	28	19	8	11	17	17	35
Numerous (>10)	23.8% (5/21)	22.9% (24/105)	40.0% (12/30)	32.1% (9/28)	78.9% (15/19)	25.0% (2/8)	0.0% (0/11)	5.9% (1/17)	23.5% (4/17)	11.4% (4/35)
Several (<10)	28.6% (6/21)	33.3% (35/105)	10.0% (3/30)	21.4% (6/28)	15.8% (3/19)	50.0% (4.8)	45.5% (5/11)	23.5% (4/17)	29.4% (5/17)	25.7% (9/35)
None	47.6% (10/21)	43.8% (46/105)	50.0% (15/30)	46.4% (13/28)	5.3% (1/19)	25.0% (2/8)	54.5% (6/11)	70.6% (12/17)	47.1% (8/17)	62.9% (22/35)
Ratios of petechial hemorrhage after changing position										
Numerous (>10)	28.6% (6/21)	27.6% (29/105)	40.0% (12/30)	32.2% (9/28)	89.5% (17/19)	37.5% (3/8)	0.0% (0/11)	11.8% (2/17)	35.3% (6/17)	14.3% (5/35)
Several (<10)	28.6% (6/21)	33.3% (35/105)	16.7% (5/30)	28.5% (8/28)	5.3% (1/19)	37.5% (3/8)	45.5% (5/11)	17.6% (3/17)	29.4% (5/17)	28.6% (10/35)
None	42.8% (9/21)	39.0% (41/105)	43.3% (13/30)	39.3% (11/28)	5.3% (1/19)	25.0% (2/8)	54.5% (6/11)	70.6% (12/17)	35.3% (6/17)	57.1% (20/35)
Rates of increases in petechial hemorrhage after changing position										
	14.3% (3/21)	13.3% (14/105)	20.0% (6/30)	7.1% (2/28)	31.6% (6/19)	37.5% (3/8)	9.1% (1/11)	11.8% (2/17)	29.4% (5/17)	11.4% (4/35)

The rates of numerous and several petechiae after changing the position of the body were high for asphyxia (89.5% and 5.3%, respectively) and low for hypothermia, hyperthermia and deaths caused by other natural causes. Other causes of death, such as sharp instrument injury, blunt force trauma, fire, intoxication, drowning, and acute heart failure, were associated with moderate rates of petechiae.

**Fig. 1:**
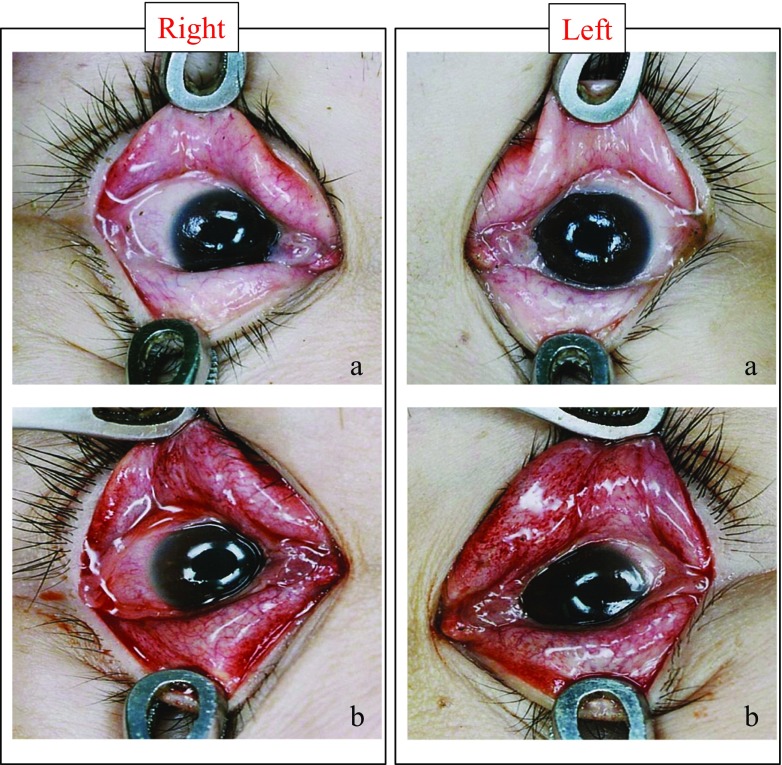
Effects of body position on palpebral conjunctival petechiae. Palpebral conjunctival petechiae typical of 30 females who died by drowning and were discovered supine (**a**), then examined while prone during autopsy (**b**)

Figure 1 and Table 5 present the rates of petechiae appearance after death after changing the positions of bodies that had sustained sharp instrument injury (14.3%), blunt force trauma (13.3%), fire (20.0%), intoxication (7.1%), asphyxia (31.6%), drowning (37.5%), hypothermia (9.1%), hyperthermia (11.8%), acute heart failure (29.4%) and other natural causes (11.4%).

Petechiae appeared in the bodies of individual aged >5 years who had sustained blunt force trauma (40.0%) and those who had died of natural causes (10.0%). However, petechiae did not appear after death when the position was changed (Table 6).

**Table 6:** Changes in palpebral conjunctival petechiae according to cause of death in children aged <5 years and found in supine position

Cause of death	Sharp instrument injury	Blunt force trauma	Fire	Intoxication	Asphyxia	Drowning	Hypothermia	Hyperthermia	Acute heart failure	Natural causes (others)
Ratios of petechial hemorrhage before changing position (*n* = 18)										
N	0	5	0	0	2	0	0	0	1	10
Numerous (>10)	–	0% (0/5)	–	–	0% (0/2)	–	–	–	0% (0/1)	0% (0/10)
Several (<10)	–	40.0% (2/5)	–	–	0% (0/2)	–	–	–	0% (0/1)	10.0% (1/10)
None	–	60.0% (3/5)	–	–	100% (2/2)	–	–	–	100% (1/1)	90.0% (9/10)
Ratios of petechial hemorrhage after changing position										
Numerous (>10)	–	0% (0/5)	–	–	0% (0/2)	–	–	–	0% (0/1)	0% (0/10)
Several (<10)	–	40.0% (2/5)	–	–	0% (0/2)	–	–	–	29.4% (0/1)	10.0% (1/10)
None	–	60.0% (3/5)	–	–	100% (2/2)	–	–	–	100% (1/1)	90.0% (9/10)
Rates of increases in petechial hemorrhage after changing position										
	–	0% (0/5)	–	–	0% (0/2)	–	–	–	0% (0/1)	0% (0/10)

### Appearance ratios of petechiae caused by cardiopulmonary resuscitation

Cardiopulmonary resuscitation was applied to 79 (27.1%) of the 291 individuals aged >5 years and to 8 (44.4%) of the 18 bodies of children aged <5 years. The ratios of numerous, several and absent petechiae appearing before changing position were 26.6% (21/79), 27.8% (22/79) and 45.6% (36/79) in those aged >5 years, and 0% (0/8), 25.0% (2/8), and 75.0% (6/8) in those aged <5 years, respectively.

### Statistical analysis

The probability that petechiae would reappear with positional conversion was significant, particularly within 1.5 days of death (OR, 2.013–2.855; CI, 95%; *p* < 0.0001) and with a BMI ≥ 25.0 (OR, 2.016–3.629; CI, 95%; *p* < 0.05). The likelihood of change was greater after asphyxia and drowning compared with other causes of death (OR, 0.046–0.369; CI: 95%, *p* < 0.05). Cardiopulmonary resuscitation was not a statistically significant factor.

## Prone position

Among 133 (30.1%) of the 442 autopsied bodies that were discovered in the prone position, 123 were aged >5 years and 10 were aged <5 years (Tables 1 and 2). The frequency of petechiae was higher among bodies found prone than supine (39.8% vs. 24.6%).

### Ratios of petechiae according to postmortem period

Based on postmortem periods of <12, 12–24, >24–36, >36–48, >4 8–60, >60–72, and > 72 h, petechiae appeared in about 16.7% (1/6), 21.9% (7/32), 9.1% (3/33), 5.9% (1/17), 12.5% (1/8), 20.0% (2/10), and 0.0% (0/19), respectively. Petechiae became obvious within 1.5 days, compared with >1.5 days postmortem. Petechiae did not appear in the bodies of children aged <5 years.

### Ratios of petechiae according to BMI and Kaup index according to age > 5 and < 5 years, respectively

We classified BMI <18.5 (*n* = 33) as malnutrition, BMI 18.5 to <25.0 (*n* = 61) as normal weight, BMI 25.0–30.0 (*n* = 24) as overweight, and BMI > 30.0 (*n* = 5) as obesity. Based on BMI, the ratios of numerous, several, and no petechiae appearing before and after changing position were as follows: malnutrition: absent 42.4% vs. 42.4%; standard: numerous 41.0% vs. 45.9%, overweight: 58.3% vs. 75.0%; obesity: several 60.0% vs. 60.0% (Table 7).

**Table 7:** Changes in palpebral conjunctival petechiae according to body mass index in bodies found in prone position

Ratios of petechial hemorrhage before changing position (*n* = 123)				
BMI	<18.5	18.5- < 25.0	25.0–30.0	>30.0
N	33	61	24	5
Numerous (>10)	33.3% (11/33)	41.0% (25/61)	58.3% (14/24)	40.0% (2/5)
Several (<10)	24.2% (8/33)	31.1% (19/61)	25.0% (6/24)	60.0% (3/5)
None	42.4% (14/33)	27.9% (17/61)	16.7% (4/24)	0.0% (0/5)
Ratios of petechial hemorrhage after changing position				
Numerous (>10)	36.4% (12/33)	45.9% (28/61)	75.0% (18/24)	40.0% (2/5)
Several (<10)	21.2% (7/33)	27.9% (17/61)	8.3% (2/24)	60.0% (3/5)
None	42.4% (14/33)	26.2% (16/61)	16.7% (4/24)	0.0% (0/5)
Rates of increases in petechial hemorrhage				
	9.0% (3/33)	9.8% (6/61)	25.0% (6/24)	0.0% (0/5)

The rates at which petechiae increased under conditions of BMI < 18.5, 18.5 to <25.0, 25.0–30.0 and > 30.0 were 9.0% (3/33), 9.8% (6/61), 25.0% (6/24), and 0.0% (0/5), respectively.

We classified those aged <5 years with Kaup indexes <13 as malnutrition (*n* = 3), 13 to <15 as thin (*n* = 2), 15 to <19 as normal weight (*n* = 5) and > 19 as overnutrition (*n* = 0). Numerous (33.3%; 1/3) and several (33.3%; 1/3) petechiae appeared in those classified as malnutrition. However, petechiae did not change after changing position (Table 8).

**Table 8:** Changes in palpebral conjunctival petechiae according to Kaup Index in children aged <5 years and found in prone position

Ratios of petechial hemorrhage before changing position (*n* = 10)				
Kaup Index	<13.0	13.0- < 15.0	15.0–19.0	>19.0
N	3	2	5	0
Numerous (>10)	33.3% (1/3)	0% (0/2)	0% (0/5)	–
Several (<10)	33.3% (1/3)	0% (0/2)	0% (0/5)	–
None	33.3% (1/3)	100% (2/2)	100% (5/5)	–
Ratios of petechial hemorrhage after changing position				
Numerous (>10)	33.3% (1/3)	0% (0/2)	0% (0/5)	–
Several (<10)	33.3% (1/3)	0% (0/2)	0% (0/5)	–
None	33.3% (1/3)	100% (2/2)	100% (5/5)	–
Rates of increases in petechial hemorrhage				
	0% (0/3)	0% (0/2)	0% (0/5)	–

### Ratios of petechiae according to cause of death

Table 9 shows that the rates at which numerous and several petechiae appeared before and after changing the position of the body were 66.7% vs. 77.8% and 22.2% vs. 11.1% for asphyxia and 61.5% vs. 61.5% and 23.1% vs. 23.1% for fire, respectively. No petechiae appeared at any time for sharp instrument injury.

**Table 9:** Changes in palpebral conjunctival petechiae according to cause of death in individuals aged >5 years and found in prone position

Cause of death	Sharp instrument injury	Blunt force trauma	Fire	Intoxication	Asphyxia	Drowning	Hypothermia	Hyperthermia	Acute heart failure	Natural causes (others)
Ratios of petechial hemorrhage before changing position (*n* = 123)										
N	4	36	13	17	9	13	6	3	7	15
Numerous (>10)	0.0% (0/4)	30.6% (11/36)	61.5% (8/13)	47.1% (8/17)	66.7% (6/9)	38.5% (5/13)	50.0% (3/6)	33.3% (1/3)	57.1% (4/7)	40.0% (6/15)
Several (<10)	0.0% (0/4)	27.8% (10/36)	23.1% (3/13)	23.5% (4/17)	22.2% (2/9)	38.5% (5/13)	16.7% (1/6)	66.7% (2/3)	28.6% (2/7)	36.7% (7/15)
None	100.0% (4/4)	41.7% (15/36)	15.4% (2/13)	29.4% (5/17)	11.1% (1/9)	23.1% (3/13)	33.3% (2/6)	0% (0/3)	14.3% (1/7)	13.3% (2/15)
Ratios of petechial hemorrhage after changing position										
Numerous (>10)	0.0% (0/4)	33.3% (12/36)	61.5% (8/13)	58.8% (10/17)	77.8% (7/9)	61.5% (8/13)	50.0% (3/6)	33.3% (1/3)	71.4% (5/7)	40.0% (6/15)
Several (<10)	0.0% (0/4)	25.0% (9/36)	23.1% (3/13)	11.8% (2/17)	11.1% (1/9)	15.4% (2/13)	16.7% (1/6)	66.7% (2/3)	14.3% (1/7)	53.3% (8/15)
None	100.0% (4/4)	41.7% (15/36)	15.4% (2/13)	29.4% (5/17)	11.1% (1/9)	23.1% (3/13)	33.3% (2/6)	0% (0/4)	14.3% (1/7)	6.7% (1/15)
Rates of increases in petechial hemorrhage after changing position										
	0.0% (0/4)	5.6% (2/36)	15.4% (2/13)	17.6% (3/17)	10.0% (1/10)	23.1% (3/13)	0.0% (0/6)	0.0% (0/3)	14.3% (1/7)	20.0% (3/15)

Table 9 presents the rates at which the incidence of petechiae increased after death when changing the position of individuals who died of sharp instrument injury (0.0%), blunt force trauma (5.6%), fire (15.4%), intoxication (17.6%), asphyxia (10.0%), drowning (23.1%), hypothermia (0.0%), hyperthermia (0.0%), acute heart failure (14.3%) and other natural causes (20.0%).

Petechiae among bodies of children aged <5 years at the time of death were numerous in one (100%) who died of blunt force trauma and in 1 (16.7%) of 6 who died of natural causes. However, changing the position of the body did not affect the appearance of petechiae after death (Table 10).

**Table 10:** Changes in palpebral conjunctival petechiae according to cause of death in children aged <5 years and found in prone position

Cause of death	Sharp instrument injury	Blunt force trauma	Fire	Intoxication	Asphyxia	Drowning	Hypothermia	Hyperthermia	Acute heart failure	Natural causes (others)
Ratios of petechial hemorrhage before changing position (*n* = 18)										
N	0	1	0	1	0	0	0	1	1	6
Numerous (>10)	–	100% (1/1)	–	0% (0/1)	–	–	–	0% (0/1)	0% (0/1)	0% (0/6)
Several (<10)	–	0% (0/1)	–	0% (0/1)	–	–	–	0% (0/1)	0% (0/1)	16.7% (1/6)
None	–	0% (0/1)	–	100% (1/1)	–	–	–	100% (1/1)	100% (1/1)	83.3% (5/6)
Ratios of petechial hemorrhage after changing position										
Numerous (>10)	–	100% (1/1)	–	0% (0/1)	–	–	–	0% (0/1)	0% (0/1)	0% (0/6)
Several (<10)	–	0% (0/1)	–	0% (0/1)	–	–	–	0% (0/1)	0% (0/1)	16.7% (1/6)
None	–	0% (0/1)	–	100% (1/1)	–	–	–	100% (1/1)	100% (1/1)	83.3% (5/6)
Rates of increases in petechial hemorrhage after changing position										
	–	0% (0/1)	–	0% (0/1)	–	–	–	0% (0/1)	0% (0/1)	0% (0/6)

### Ratios of petechiae according to cardiopulmonary resuscitation

Cardiopulmonary resuscitation was applied to 19 of 123 (15.4%) deaths. The rates of numerous, several, and no petechiae appearing before changing body position were 42.1% (8/19), 31.6% (6/19) and 26.3% (5/19), respectively. Cardiopulmonary resuscitation was not applied to children aged <5 years (Table 2).

### Statistical analysis

Statistical analysis revealed no significant probability of petechiae reappearing due to positional conversion in terms of postmortem period, BMI, cause of death, or cardiopulmonary resuscitation.

## Discussion

Postmortem experiments on stillborn children conducted by Haberda in 1898 revealed the development of position-induced conjunctival petechiae [8]. During the latter half of the twentieth century Reh and Haarhof observed petechiae and more intensive subconjunctival bleeds in bodies that were positioned with the head down for periods ranging from 5 to 47 h [9]. Petechiae are frequently found in the conjunctivae and eyelids of prone bodies during autopsy [5]. A recent experimental study found that conjunctival petechiae develop postmortem when the head is placed below the body for several hours [7].

The present study found a higher frequency of numerous petechiae in bodies that had been discovered in the prone, compared with the supine position (39.8% vs. 24.6%). However, the ratio of increased amounts of petechiae was higher after changing the body from the supine, to the prone position (numerous, 24.6%–28.8%; several, 26.8%–27.2%). The rates at which petechiae increased in the prone position did not significantly differ among all factors. One explanation for this is the large amount of extant petechiae by the time a body was discovered in the prone position. In contrast, the amount of petechiae obviously changed in bodies that had been discovered in the supine position <1.5 days, compared with >1.5 days postmortem (<12, 12–24 > 24–36 h: 38.5%, 21.4% and 17.2%, respectively). The assumed explanation for this finding is similar to the spread of postmortem lividity [10], in which the movement of blood in vessels becomes difficult due to gravity, and hemoglobin leaks through blood vessels along with liquid components due to hemolysis [11, 12].

Petechiae appeared more frequently in obese bodies than in those with a lower BMI. The tendency towards an increased rate of petechiae due to changes in body position was higher in obesity compared with a lower BMI which might be associated with larger circulating blood volumes [13], and increased abdominal venous pressure with positional changes [14, 15]. However, this phenomenon was not clear in children aged <5 years.

The prevalence of petechiae and the rate at which they increased with positional changes were very high after asphyxia and drowning, and low after death due to hypothermia, hyperthermia and natural causes other than acute heart failure. Blood fluidity in the supine position might have influenced the rate at which petechiae increased in death due to asphyxia and drowning, being higher for acute death than after hypothermia and hyperthermia with subacute death/delayed death. These findings suggested that hypostatic blood can become redistributed in the face soon after death and partly involve the appearance of, or increase in the amount of petechiae in the palpebral conjunctivae. This phenomenon might reflect microvascular injury during distress before death caused by arteriolar hyperemia, vascular congestion and tissue hypoxia [3]. However, the postmortem appearance of petechiae in the palpebral conjunctivae is more likely to indicate the predominant influence of systemic hypoxia on microvessels without bleeding at the time of death, which can also be related to petechiae involved in postmortem hypostasis.

Cardiopulmonary resuscitation has repeatedly been discussed as a possible cause of conjunctival petechiae [16, 17], but one study has found that cardiopulmonary resuscitation is not a cause of conjunctival hemorrhage [18]. The present study found that cardiopulmonary resuscitation did not affect the appearance of petechiae when the deceased was aged >5 years.

We concluded that a postmortem period within 1.5 days, BMI ≥ 25.0, and death caused by asphyxia and drowning (acute death) are associated with positional changes in amounts of petechiae.

## Key points

1. Positional changes in petechiae occurred within 1.5 days postmortem and were associated with BMI ≥ 25.0, asphyxia and acute drowning.
2. Forensic pathologists should assess the presence of conjunctival petechiae before changing the position of a body.
3. The possibility of positional asphyxia should be carefully considered.
4. Cardiopulmonary resuscitation might influence petechiae in addition to body position at discovery.
